# Eco-products value realization and eco-industry design of afforestation in the Karst Desertification Control

**DOI:** 10.1371/journal.pone.0321541

**Published:** 2025-04-28

**Authors:** Yu Zhang, Zefu Zhang, Kangning Xiong, Youze Ning

**Affiliations:** 1 Tangshan Key Laboratory of Simulation of Urban Ecosystem, Department of Resource Management, Tangshan Normal University, Tangshan, People’s Republic of China; 2 Geography and Environment Sciences, Schools of Karst Science, State Engineering Technology Institute for Karst Desertification Control, Guizhou Normal University, Guiyang, People’s Republic of China; Agriculture and Forestry University, NEPAL

## Abstract

Karst desertification (KD) severely restricts rural development in China’s karst regions. To ensure both ecological security and economic sustainability, afforestation is essential for karst desertification control (KDC). This paper utilizes scientific approaches, including Ecological Product (EP) value accounting (which evaluates the value of ecosystem - provided products and services for humans) and data statistical analysis, to explore the functions of afforestation in EP distribution, value realization, and Ecological Industry (EI) formation. The study reveals several key findings. (1) In the three study areas, afforestation significantly impacts the regulation of service product values. For instance, in the Shibing area, Horsetail pine is the main EP supplier; in the langyashan study area, it is cypress; and in the Zunhua study area, oil pine. (2) In the southern karst (exemplified by the Shibing study area), EP values are influenced by factors like stand height, diameter at breast height, altitude, organic carbon, and soil total nitrogen. In the northern karst (using langyashan study area and Zunhua study areas as examples), soil total nitrogen, organic carbon, and porosity are the main determinants. (3) With decreasing altitude, industrial distribution shifts from an ecological - forest - based industry to one with co - existing ecological and economic forests, and finally to an economic - forest - based industry. Rational selection and breeding of economic forest species aid in creating ecolabel brands. This small - watershed - scale analysis improves the accuracy of assessing ecological and economic development in karst areas, providing a scientific basis for decision - making to strengthen ecological restoration and enhance people’s well - being.

## 1. Introduction

In the wave of rapid global economic and social development, human over-exploitation and utilisation of resources have inevitably triggered the destruction of the ecological environment. This has resulted in a drastic reduction in biodiversity, posing a serious threat to the long - term, sustainable development of society [1]. It is worth noting that in the past fifteen years, China has made remarkable achievements in karst desertification (KD) areas by promoting natural restoration projects and large - scale afforestation programs, significantly increasing vegetation coverage and marking a step forward in ecological “re - greening” [2]. With the acceleration of labor export, urbanization, and the promotion of ecological protection and karst desertification control (KDC) projects, the area of afforestation for KDC has grown significantly [3]. As afforestation and other ecological restoration work deepen, the results of ecological civilization construction become more prominent. However, the tension between ecological protection and sustainable economic development has emerged, which has led to the proposal and in - depth discussion of the concept of Ecological Product (EP).

Overseas scholars often use terms like “Ecosystem Services” or “Eco-Label Products” [4]. The United Nations Environment Programme (UNEP) - led Programme on Ecosystems and Biodiversity defines ecosystem services as “the direct or indirect contribution of ecosystems to human well - being” [5]. In China, the concept of Ecological Industry (EI) was initially proposed in the 2010s in central and western China’s poor ecological function areas as a solution to the contradiction between ecological construction and economic development, with the slogan of “industrialisation of ecological construction” [6]. Wen Tiejun proposed that eco-industrialisation is an effective way to achieve healthy and stable development of ecological resource advantages in a market economy environment [7]. This is consistent with the previous idea that EI can solve the contradiction between ecology and economy, further deepening the understanding of how to transform ecological advantages into economic resources. This paper defines afforestation EP as the end products or services produced by human social labor on the ecosystems in the management of KD and provided to human society for use and consumption. The afforestation Ecological Industry (EI) is an industrial model that ecologically exploits and utilises the economic value of afforestation forests to create a virtuous cycle.

Current research on realising the value of EP in ecologically fragile areas is insufficient in economic, policy, and social aspects [8]. The EP supply capacity of forests is vulnerable to decline due to deforestation and degradation, and there is a great deal of effort to maintain the EP supply needs of forests [9]. Since industrial assistance is crucial for poverty alleviation, it is important to develop local EI with special characteristics according to local conditions [10]. Internationally, classic EP value estimation methods such as market value and conditional value have been formed, along with assessment methods using remote sensing, geographic information systems, and the InVEST model [11–13]. However, in general, current EP value accounting indicators in various regions have difficulty accurately measuring the value of EP. As forest management focuses on ecological service products [14–18], driving the formation of EI with the realisation of EP values and transforming forest ecological advantages into economic resources has become an urgent issue [19]. Since the beginning of the last century, China has implemented large - scale afforestation and greening projects, leading to the rapid development of forest resources. But there is a lack of research on the sustainable management of forest ecosystems, and exploring the synergistic development of ecological resources restoration and eco-industrialisation is an urgent taskd [20]. Currently, the advantages and potentials of various regions in EI have yet to be tapped. There is a general lack of research on eco-industrialisation and industrial eco-ecological economic systems, as well as a lack of market recognition of EP [21].

**Fig 1:**
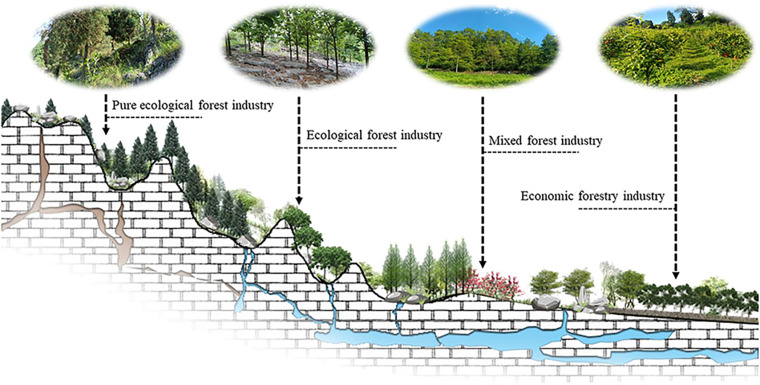
Design of ecological industry layout for afforestation and supply enhancement of ecological products in the Karst Desertification Control.

EP value realisation is crucial for promoting ecological protection, economic development, and maintaining a balance between the two [22]. Afforestation in KD areas aims to achieve ecological and economic benefits. However, high mountain slopes, steep slopes, fragmented land resources, and decentralized smallholder management make it difficult to form the EI of afforestation. Exploring the main body of responsibility for EP is the key to EI development [23]. Drawing on the concept of natural management, developing high-quality tree germplasm resources, selecting and breeding high-quality varieties of native tree species in KDC areas, gradually forming a stable supply of EP, and establishing a diverse and sustainable forest EI are the core of breaking through the situation of low supply and industrialisation difficulties (Fig 1).

In view of the high ecological vulnerability and rapid development of afforestation in KDC, and in response to the cutting-edge scientific problems of enhancing the ecological stability of afforestation in KDC and the key technical challenges of ecological value development, this study combines the strategic and scientific and technological needs of pursuing the economic value of ecological restoration and ecological protection in global karst areas with the innovative adoption of the EP value accounting method and the mathematical statistical analysis method. Through in-depth research on the mechanism of the impact of afforestation on soil, we aim to reveal the mechanism of realising the value of EP from afforestation, and then break through the bottleneck of value realisation driving the design of EI. On this basis, we constructed an ecological value accounting model and explored the realisation path of EP value. These research results not only provide a scientific basis for the realisation of EP value in KDC, but also provide an important reference for the formation and design of EI, thus promoting the integration of KDC and sustainable development goals.

## 2. Study area and methods

### 2.1. Study area selection

**Fig 2:**
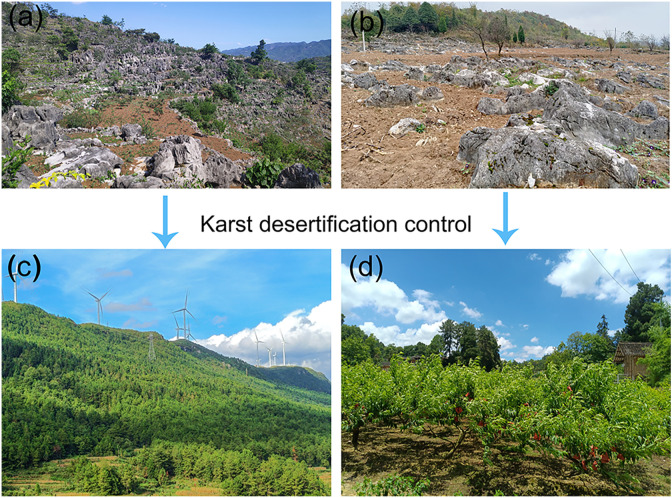
Karst Desertification Control and artificial afforested landscape. (a) Karst desertification in large slope area; (b) Karst desertification in gentle slope area; (c) Ecological forest; (d) Economic forest.

Artificial forestation for KDC is an important part of the main city of China’s forests. In the KDC area, ecological forest is based on according to different ecological location conditions, in the ecological location of important slope (more than 15 °) and soil conditions allow land, select adaptable and suitable native tree species construction of afforestation, used to maintain soil and water and promote ecological restoration; economic forest is in the slope of the land with gentle (less than 15 °), easy access, select suitable for local villages of high-quality, high-yield, Economic forests are afforestations constructed on land with gentle slopes (below 15°) and easy access, in which high-quality, high-yield and high-efficiency economic forest species suitable for local villages are selected to improve the ecological environment and increase the economic income of the inhabitants. As an important means of ecological management and economic development in KD areas, afforestation forests (Fig 2) have already achieved fruitful results [24–27].

**Fig 3:**
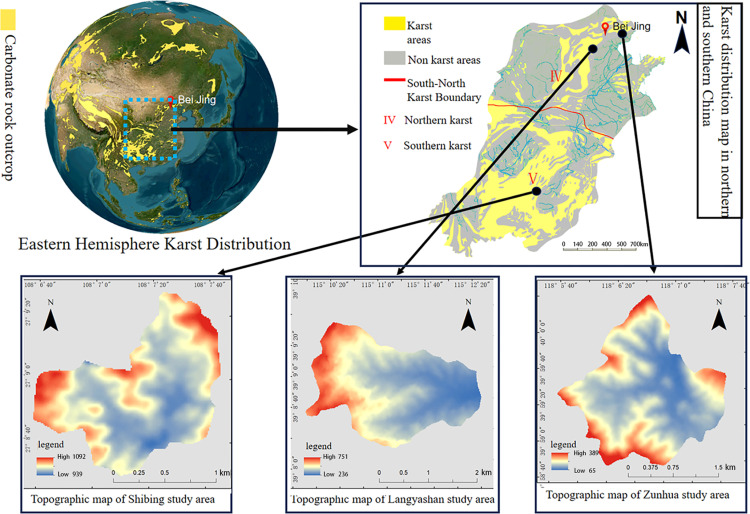
Study area location map. Source: Jilin-1 satellite image data (https://www.jl1mall.com/lab/).

Due to the special conditions of lithology, geological structure, climate, hydrology and neotectonic movement, the karst formations in southwestern China are characterised by peaks and forests, closed depressions, deep and sharp dissolution scars, laterite, surface calcareous China, huge cave systems, underground river systems, and a large number of secondary carbonate deposits in caves; while the karsts in semi-arid areas of northern China are characterised by normal mountains, box-frost residual chert pinnacles, limestone corners, shallow and fine dissolution scars, dry valleys, large springs, small caves, and a small number of secondary carbonate deposits in caves [28]. It is of great significance to study the management of KD in the south and north and the realisation of ecological value after the management, to better promote the research on the mechanism of realising the value of EP in different landforms and climatic zones of China’s karst, and to promote the construction of a beautiful China. According to the principles of typicality, representativeness and demonstration, we selected the Shi Bing study area in the southern karst and the Langyashan study area and Zunhua study area in the northern karst (Fig 3). Among them, Shi Bing study area represents the afforestation area under the ecological protection strategy of KDC, Wolf’s Tooth study area is the afforestation area under the ecological protection-restoration strategy of KDC, and Zunhua study area is the afforestation area under the ecological restoration strategy of KDC. The selection of these three study areas (The field sampling site chosen for this study is a publicly accessible area and does not necessitate any associated permits.) as research objects can not only represent the development direction of EP and EI of afforestation after KDC under different ecological strategies of karst, but also represent the direction of ecological and economic development of afforestation under KDC of karst in China.

#### (1) Heichong Sub-watershed Study Area in the Buffer Zone of the Shi Bing Karst World Natural Heritage Site (abbreviated as “Shi Bing Study Area”).

The Shibing Karst is in Shibing County, eastern Guizhou Province, at the transition from the Yunnan - Guizhou Plateau’s eastern edge to western Hunan’s low hills (the second to third - level terrain transition in China). It has a subtropical humid monsoon climate and lies in the middle subtropical karst canyon area, with a north - high and south - low terrain, mainly featuring peaks and canyons. The soil is mainly thin - layer limestone soil from dolomite weathering, and it’s a World Natural Heritage Site with high forest coverage.

The Heichong sub - watershed, in the southeast buffer zone of the Shibing natural heritage site (108°11’52“ - 108°13’34” E, 27°13’56” - 27°15’42” N), covers 178.07 hectares at an altitude of 978 m, with an average annual temperature of 18 °C. Despite being a karst area, it has less soil loss due to proper protection, relatively good soil conditions as most cultivated land is in depressions and there’s little slope open land. The sub - watershed has mostly tall - tree forests, mainly coniferous, mixed coniferous - broad - leaved, and broad - leaved evergreen forests, with high forest coverage and good forest connectivity.

#### (2) Langyashan sub-watershed study area in the Langyashan National Forest Park, Yixian County (referred to as the “Langyashan study area”).

The Langyashan sub - watershed study area is in Baoding City, Hebei Province, North China, at the mountain - plain intersection in western Yixian County. Its coordinates are (115°17’12“ - 115°21’26″E, 39°13’72″ - 39°16’04 “N), covering 537.6 hectares. It has a warm - temperate semi - moist monsoon climate, with dry, windy springs and concentrated rainfall in hot summers. The average altitude is 320m. It lies at the eastern foot of the northern Taihang Mountains, with complex geomorphology mainly composed of pure or greyish dolomite. The area has mainly medium and small trees, with coniferous and deciduous broad - leaf forests, and a low forest cover.

#### (3) Huangtuling sub-watershed study area of Zunhua National Three-North Protective Forest Project Area (referred to as “ Zunhua study area”).

The Huangtuling sub - watershed study area is in Zunhua City, Hebei Province, North China. It’s at the northeast of Hebei, bordering Qinhuangdao to the east, Beijing to the west, Tangshan to the south and Chengde to the north. Coordinates are (118°09′82″ - 118°13′11″E, 39°97′95″ - 40°00′58″N), covering 436.2 ha.

It has a temperate semi - moist monsoon climate, with an average altitude of 103 m, average annual temperature of 10.9°C and annual precipitation of 724.7 mm. Situated at the end of the Yanshan Mountain Range, its geomorphology is well - developed and mainly consists of pure or greyish dolomite. The area has mainly medium and small trees, with vegetation types like coniferous, deciduous broad - leaf and mixed coniferous - broad - leaf forests, and a relatively low forest coverage rate.

### 2.2. Technological route

**Fig 4:**
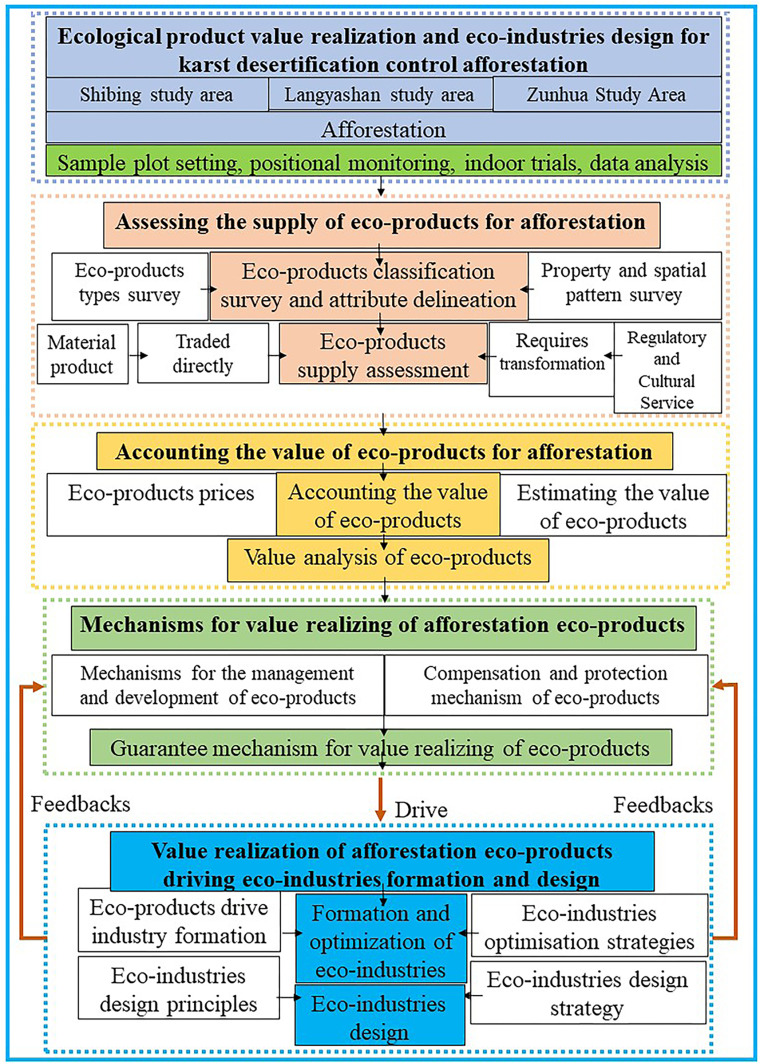
Technology roadmap.

Based on the core theories of human-land co-ordination, ecosystem diversity and circular economy in the fields of geography, ecological economics and landscape architecture, this study focuses on the core scientific issues and technological needs, such as human-land conflicts, insufficient structural stability of afforestation ecosystems, and weakening of the service capacity in barren habitats in karstic KD areas. To address these challenges, we selected the Huangtuling subwatershed and the Langyashan subwatershed in the mountainous areas of Zunhua and Yixian in the northern dolomite karst ecosystem type, and the Shibing Karst Heichong subwatershed in the mountainous area of Shibing in the southern dolomite karst ecosystem type, as our study areas. Between 2021 and 2024, we systematically collected data on dominant plant species and soils through continuous positional observations at 54 experimental sample plots. Combining various research methods, such as human-computer interactive remote sensing interpretation method, field positioning monitoring and indoor experimental validation method, EP value accounting method, and data statistical analysis method, we thoroughly explored the frontier scientific issues of dominant function forest product value accounting and EP supply accounting of afforestation for KDC under different ecological development strategies. At the same time, we are also committed to the research and development of common key technologies, with a view to advancing the systematic research on the driving mechanism of the value realisation of afforestation products on the formation of EI. This study not only provides a scientific basis for KDC, but also provides strong support for the sustainable development of EI (Fig 4). Through this comprehensive interdisciplinary study, we expect to make substantial contributions to solving the human-land conflicts in KD areas, enhancing the stability and service capacity of ecosystems, as well as promoting the realisation of the value of EP and the process of eco-industrialisation.

### 2.3. Research method

#### (1) Human-computer interactive remote sensing interpretation method.

USGS (https://earthexplorer.usgs.gov/) was selected to acquire Landsat8 satellite remote sensing image data from 2021–2024 in the three study areas of Zunhua, Wolverine Mountain and Shibing, to extract data on vegetation cover, soil cover, rock exposure rate, slope and spatial distribution of afforestation landscapes.

#### (2) Field positioning monitoring and indoor experiment verification method.

The afforestation sample plots were selected through comprehensive field investigation, and were divided into two types of ecological forests and economic forests according to the purpose of afforestation production and afforestation. Data collection for the two types of sample plots were carried out separately, mainly soil chemical soil sampling, soil physical soil sampling, forest basic information survey, EP species survey, and EP attribute division.

#### (3) Ecological product value accounting method.

Using mathematical models to analyse the dominant functional EP of afforestation derived from field surveys and experiments, and carry out EP value accounting, qualitative and quantitative analysis of EP, to provide direct data support for the realisation of EP value and EI design. Based on the objectives of this study, combining the “Specification for the Assessment of Forest Ecosystem Service Function (GB/T 38582-2020)”, “Technical Guidelines for the Accounting of Gross Terrestrial Ecosystem Product (GEP)”, “Specification for the Accounting of Gross EP Value” and other information and Xie Gao Di et al. [29] Equivalent Factor Method to select the supply and value of EP of afforestation for the research content, combined with the special geological and geomorphological background of the north and south karst regions, clarified the differentiation between economic forests and ecological forests, and condensed the accounting indexes of EP of afforestation (Table 1).

**Table 1:** Indicator system for accounting of ecosystem production value of afforestation.

Product Category	Code	Accounting subjects				Indicator of effectiveness	Unit
		Level 1 indicators	Code	Level 2 indicators	Code		
Physical Products	A	Forestry Products	A01	Warp fruit yield	A0101	Warp Fruit Yield	t
				Fruit value	A0102	Warp Fruit Value	Yuan
Regulatory services	B	Soil Conservation	B01	Soil fertility retention	B0101	Nitrogen retention	t
					B0102	Maintaining phosphorus content	t
					B0103	Maintaining Potassium Content	t
					B0104	Maintaining organic matter content	t
				Soil fertility retention value	B0105	Maintaining and increasing nitrogen, phosphorus and potassium organic matter values	Yuan
		Water Conservation	B02	Physical volume of water retention	B0201	Amount of Water Nourishment	t
				Value of water conservation	B0202	Value of water resources	Yuan
		Atmospheric purification	B03	Amount of air pollutants purified	B0301	Sulphur dioxide purification capacity	t
						Nitrogen oxide purification capacity	t
						Particulate matter purification capacity	t
				Atmospheric purification value	B0303	Sulphur dioxide purification value	Yuan
						Value of purifying nitrogen oxides	Yuan
						Value of purifying particulate matter emissions	Yuan
		Carbon Sequestration	B04	Carbon sequestration	B0401	Total amount of CO2 fixed	t
				Value of carbon dioxide fixation	B0402	Value of CO2 fixation	Yuan
		Climate regulation	B05	Climate regulation value	B0501	Vegetation evapotranspiration	mm
					B0502	Climate regulation value	Yuan
Cultural services	C	Equivalent Shadowing	C01	Equivalent value	C0101	Value of cultural services	Yuan

#### (4) Data statistical analysis method.

Research methods such as statistics, econometric geography, landscape ecology, etc. were applied to analyse the experimental data obtained from the images and sample plots using software such as SPSS 22.0, Microsoft Excel 2010, Origin 2018, and so on. Statistics on the distribution of landscape patterns of afforestation ecosystems were used to determine the main influencing factors of EP supply in afforestation ecosystems through factor analysis and principal component analysis.

#### (5) Ethics statements.

This study does not involve human or animal experiments and is based solely on public data/theoretical analysis/mathematical modeling, therefore it does not involve any ethical issues.

## 3. Results

### 3.1. Ecological product value of afforestation in karst desertification control

#### 3.1.1. Ecological product value of afforestation.

**(1) Value of ecological products in different supply modes:** From the perspective of total EP value (Table 2), the total EP value of afforestation in Shibing study area is 45.8729 million yuan, from the perspective of supply mode, the value of material product is 176.6 thousand yuan, accounting for 0.39% of the total value, the value of regulating service product is 45.450 million yuan, accounting for 99.08% of the total value, and the value of cultural service product is 246.3 thousand yuan, accounting for 0.54% of the total value. 0.54 per cent. The total value of afforestation EP in the Langyashan study area was 47,434,500 yuan, the value of material products was 1,576,600 yuan, accounting for 3.32% of the total value, the value of regulating service products was 45,711,900 yuan, accounting for 96.37% of the total value, and the value of cultural service products was 145,900 yuan, accounting for 0.31% of the total value. The total value of EP of afforestation in the Zunhua study area was 47,842,600 yuan, the value of material products was 5,321,700 yuan, accounting for 11.12 per cent of the total value, the value of regulating service products was 42,382,900 yuan, accounting for 88.59 per cent of the total value, and the value of cultural service products was 138,000 yuan, accounting for 0.29 per cent of the total value. From the proportion of EP value in each study area, it can be seen that the proportion of material products in Shibing and Langyashan study areas is less than that in Zunhua study area, and the ability of direct realisation of EP is weaker. The value of regulating service products is the highest proportion in the Shi Bing study area, which basically represents the value of EP of all afforestation in this study area.

**Table 2:** Value of ecological products in the study area.

Research Areas	Share of value of material goods (%)	Value share of regulating service products (%)	Value share of cultural service products (%)	Total value of eco-products (million yuan)
Shibing Study Area	0.39%	99.08%	0.54%	4587.29
Langyashan Area	3.32%	96.37%	0.31%	4743.45
Zunhua Study Area	11.12%	88.59%	0.29%	4784.26

**(2) Comparison of ecological product value of afforestation of different functional types:** From the perspective of afforestation forest attributes (Table 3), the EP value of ecological forests in Shibing study area is 45,872,900 yuan, accounting for 97.57% of the value of afforestation EP in this study area; the EP value of economic forests is 1,113,600 yuan, accounting for 2.43% of the value of afforestation EP in this study area. The EP value of ecological forests in the Langyashan study area was 40,797,800 yuan accounting for 86.01% of the EP value of afforestation in this study area; the EP value of economic forests was 6,636,700 yuan, accounting for 13.99% of the EP value of afforestation in this study area. The EP value of ecological forests in the study area of Zunhua was RMB 34,360,000, accounting for 71.82% of the EP value of afforestation in the study area; the EP value of economic forests was RMB 13,482,600, accounting for 28.18% of the EP value of afforestation in the study area. Analysing the statistical results, it can be inferred that the economic forest industry in Zunhua study area is better developed, and its ecological restoration-type EP and EI have a clear path of development, with great potential for development.

**Table 3:** Ecological product values of afforestation for different functional forest types.

Research Areas	Functional forest type	Total value of eco-products of different functional forest types (yuan)	Percentage (%)	Total value of eco-products (million yuan)
Shibing Study Area	S Economic Forest	111.36	2.43%	4587.29
	S Ecological Forest	4475.92	97.57%	
Langyashan Area	Y Economic Forest	663.67	13.99%	4743.45
	Y Ecological Forest	4079.78	86.01%	
Zunhua Study Area	Z Economic Forest	1348.26	28.18%	4784.26
	Z Ecological Forest	3436.00	71.82%	

**Fig 5:**
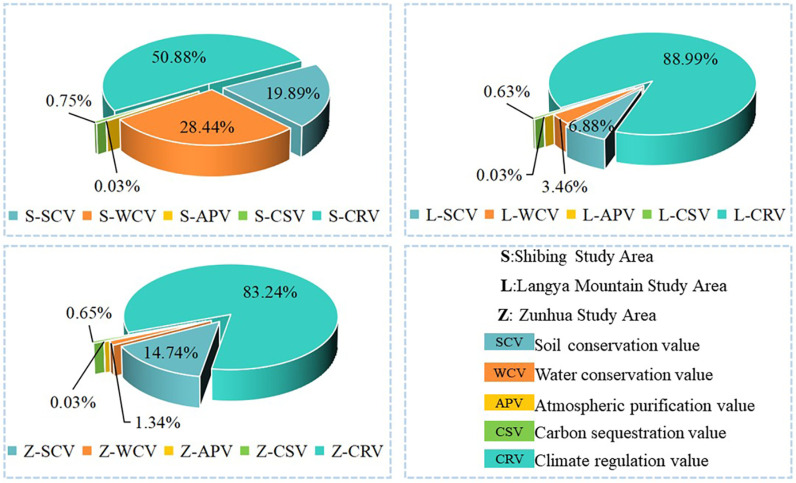
Reconciliation services product value analysis.

**(3) Comparison of the value of ecosystem regulation service functions:** Based on the ecosystem regulation service function perspective (Fig 5), the value of regulation service products in the Shibing study area was mainly contributed by soil conservation, water conservation and climate regulation, accounting for 19.89%, 28.44% and 50.88% of the total value of regulation service products, respectively, while the value of atmospheric purification and carbon sequestration services was the least, at 0.03% and 0.75%. The value of regulating service products in the Langyashan study area was contributed by climate regulation and other service products, accounting for 88.99% of the total regulating service product value, followed by soil conservation and water conservation service products, accounting for 6.88% and 3.46%, and carbon sequestration and atmospheric purification services were the lowest in value, with the sum of the two accounting for less than 1%. The value of regulatory services in the Zunhua study area is mainly contributed by the value of soil conservation and climate regulation services, accounting for 14.74% and 83.24%, while the value of water conservation only accounts for 1.34%, and the value of carbon sequestration and atmospheric purification services is less than 1%.

#### 3.1.2. Ecological product value per unit area of different forest types for afforestation.

**Fig 6:**
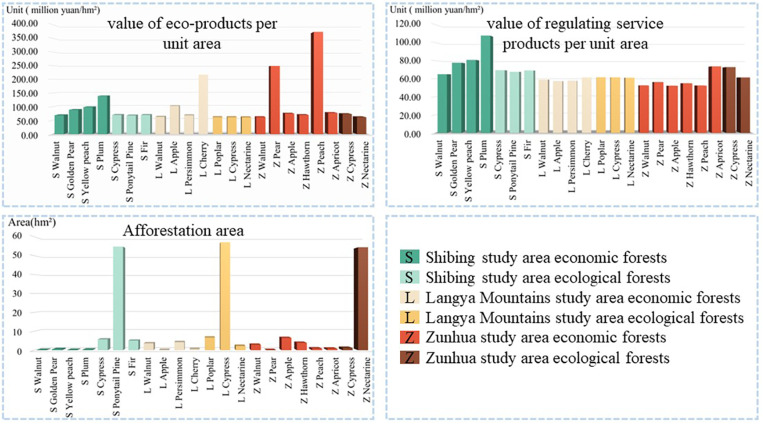
Ecological value of dominant species per unit area.

The area of afforestation in different study areas and different forest types varies greatly, and the EP values of different dominant species are difficult to provide indications for ecological and economic development. Therefore, this paper uses the EP value per unit area of afforestation for comparative analysis (Fig 6).

The value of afforestation material products due to the region, market sales methods and other impacts is not easy to do unit area comparison, afforestation cultural service products between the unit area is basically the same also does not have the comparability, so the material products and cultural service products value is not in a separate analysis of the unit area of different forest types. This paper focuses on analysing the value of regulating service products per unit area in three research areas, and exploring the direction of the development advantages of afforestation EP. Plum has the highest value of regulating service products per unit area in Shibing study area, which is 1,077,800 Yuan/hm², followed by yellow peach (808,300 Yuan/hm²), and walnut is the lowest (650,000 Yuan/hm²). Poplar has the highest value of EP regulating service products per unit area in Langyashan study area, which is 616,400 Yuan/hm², cypress is the second (615,600 Yuan/hm²), and apple is the lowest (571,800 Yuan/hm²). In the study area of Zunhua, apricot forest had the highest value of EP regulating service products per unit area of 736,600 yuan/hm², cypress was the second highest (728,300 yuan/hm²), and apple was the lowest (527,700 yuan/hm²).

The differences in these data can be attributed to multiple factors. Natural factors like climate and soil have a profound impact. For example, in the Shibing study area, plums may have a high value of regulating service products per unit area because they are well - adapted to the local climate and soil conditions. The local climate, with its specific temperature, precipitation, and humidity levels, might be highly conducive to the growth and ecological functions of plums. In contrast, walnuts have a lower value, perhaps due to less - favorable soil nutrient composition or a mismatch with the local climate pattern for optimal growth and ecological performance. In terms of human - induced factors, market orientation also plays a role. If a certain species has a high market demand for its products or ecological services, it is likely to receive more investment in management and cultivation, which can enhance its overall value. For example, if a particular tree species provides excellent carbon - sequestration services and there is a growing market for carbon - offsetting credits, efforts will be made to improve its growth conditions, thus increasing its value.

The largest area in the three study areas is ecological forest, but through analysis, it was found that ecological forests do not have the highest value of EP per unit area. This could be due to tree - species characteristics and management approaches. For instance, in the Shibing study area, horsetail pine, which has the largest area, has relatively low EP value per unit area and regulating - service - product value per unit area. This might be because horsetail pine has slow - growth rates and limited ecological functions in terms of regulating services compared to other species like plums. In addition, the management approach matters. If the management of ecological forests is mainly for conservation purposes with less - intensive cultivation and improvement measures, their productivity and value - generating capacity per unit area may be restricted. In the Langyashan study area, cypress, despite its large area, has a relatively low EP value per unit area, which may be related to improper management practices or its natural growth characteristics that limit its contribution to ecological services. In the Zunhua study area, the large - area greasewood has a low value per unit area, possibly because of a lack of appropriate management strategies to enhance its ecological functions.

These findings offer valuable insights for the development of the EI. When planning afforestation, it is crucial to consider both natural adaptability and market demand. Selecting tree species that are well - adapted to the local environment and have high market potential can maximize the ecological and economic values. Moreover, optimizing the management of different forest types, especially ecological forests, by adopting more scientific cultivation and improvement measures, can enhance their productivity and value - generating capacity per unit area.

### 3.2. Ecological product value realisation of afforestation for karst desertification control

#### 3.2.1. Controlling factors affecting the value of dominant function service products.

This section primarily analyzes the controlling factors influencing the value of dominant functional service products from afforestation in KDC. Through systematic organization and statistical analysis of relevant data (For the Shibing study area, the supporting information can be found in “S1 Table” and “S2 Table”; for the Langyashan study area, the supporting information are provided in “S3 Table” and “S4 Table”; and for the Zunhua study area, the supporting information are detailed in “S5 Table” and “S6 Table”), the contribution levels and variability of these factors to the value of service products are elucidated. The findings aim to provide a scientific basis for optimizing and enhancing the value of EP derived from afforestation in KDC areas.

**Fig 7:**
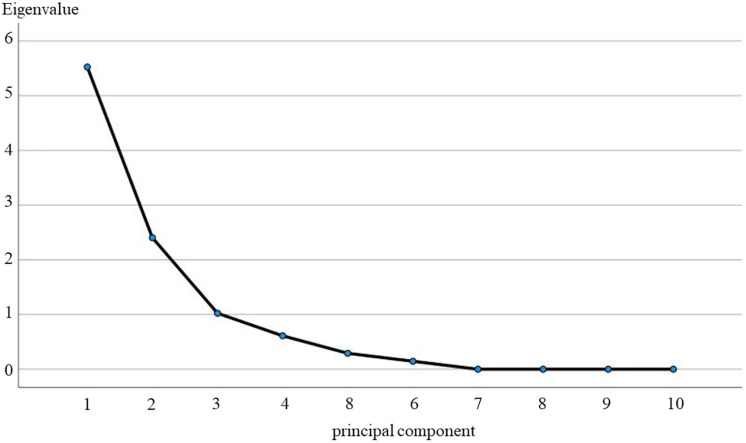
Gravel map of principal component analysis for the Shibing study area.

**(1) Southern karst master control factor analysis:** In the comprehensive analysis for the Southern Karst Shibing study area, this study fully utilises the SPP22.0 software, aiming at accurately calculating the 10 key indicator factors within the area, so as to obtain the specific contribution rate of each principal component and its cumulative effect. Table 4 and Fig 7 exhaustively demonstrate the eigenvalues and corresponding contribution rates of the principal components.

**Table 4:** Eigenvalues of the correlation coefficient matrix for the Shibing study area.

Ingredients	Eigenvalues	Contribution rate%	Accumulated contribution rate%
1	5.525	55.246	55.246
2	2.404	24.038	79.284
3	1.024	10.236	89.520
4	0.611	6.109	95.629
5	0.291	2.912	98.541
6	0.146	1.459	100.000
7	9.405E-17	9.405E-16	100.000
8	5.060E-17	5.060E-16	100.000
9	−3.55E-16	−3.55E-15	100.000
10	−3.96E-16	−3.96E-15	100.000

In the process of data dimensionality reduction, we followed the strict statistical principle of setting the eigenvalue greater than 1 and the cumulative variance contribution rate over 85% as the thresholds, so as to ensure that the selected principal components can represent the information of the original dataset to the greatest extent possible. Based on this criterion, we find that the first three principal components are particularly significant, their contribution rates are 55.25%, 24.04% and 10.24% respectively, and the cumulative contribution rate is as high as 89.52%. This result is a strong indication that these three principal components dominate the main control factors affecting the characteristics of the study area. To further validate this conclusion, we also combined the results of the analysis of the gravel plot (Fig 7). In its intuitive form, the gravel plot shows the trend of the eigenvalues of the principal components, thus providing us with strong support regarding the rationality of the selection of the principal components. Combining the graphical data and the morphology of the gravel diagram, we are convinced that the first three principal components are not only numerically significant, but also play a central role in explaining the characteristics of the study area.

After the maximum variance method of rotating the matrix (Table 5), we obtained the following key results. In the first principal component, stand height, mean diameter at breast height (Dbh), elevation, organic carbon and soil total nitrogen had significantly larger loadings, which together constituted the main characterisation of the first principal component, highlighting its central position in the dataset. In the second principal component, soil porosity, soil water content and soil total nitrogen became the main load factors, and the influence of these variables on the second principal component was particularly significant.

**Table 5:** Rotated load matrix of factors for the Shibing study area.

Factor	Ingredients		
1	2	3
Soil total phosphorus	−0.931	0.163	0.097
Stand height	0.910	0.110	0.346
Average diameter at breast height	0.898	0.223	0.290
Elevation	0.735	−0.087	0.437
Soil porosity	−0.002	0.978	0.007
Soil water content	−0.084	0.974	0.021
Soil bulk weight	−0.505	−0.731	−0.405
Soil potassium	−0.086	0.049	−0.894
Soil organic carbon	0.601	0.288	0.666
Soil nitrogen	0.540	0.347	0.661
Variance contribution/%	55.246	24.038	10.236
Cumulative contribution/%	55.246	79.284	89.520

Further analyses showed that the loadings of soil total phosphorus, stand height, average diameter at breast height (ADB), elevation, soil organic carbon (SOC) and soil total nitrogen (TN) were also more prominent in the third principal component. Particularly noteworthy is that in the first principal component, stand height and average diameter at breast height had the highest loadings of 0.91 and 0.90, respectively, which showed the high correlation between these two variables in the first principal component and their importance. In the second principal component, soil porosity and soil water content had the highest loadings of 0.98 and 0.97, respectively, which further emphasised the dominant role of these two variables in the second principal component. And in the third principal component, the loadings of soil organic carbon and soil total nitrogen were both 0.66, indicating that these two variables also occupied a more important position in the third principal component.

**Fig 8:**
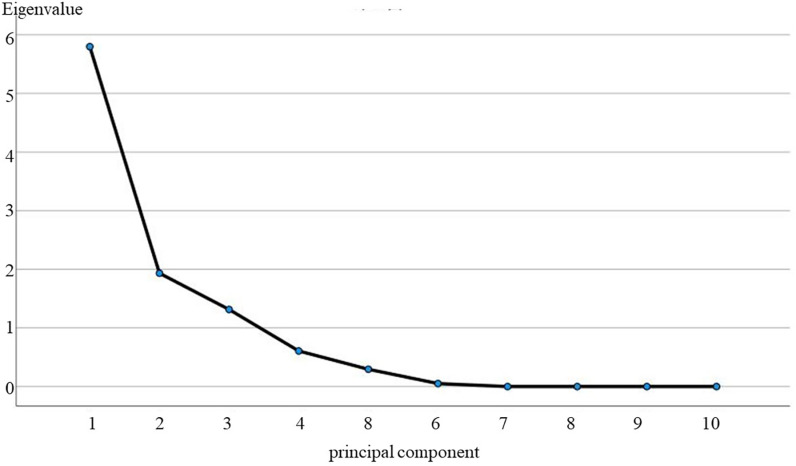
Gravel map of principal component analysis for the Langyashan study area.

**(2) Northern karst controlling factor analyses:** Using SPP22.0 software to calculate the 10 indicator factors in the study area of Langyashan in the northern karst, aiming at obtaining the contribution rate of each principal component and the cumulative contribution rate, in which the eigenvalues and contribution rates of the principal components are shown in Table 6 and Fig 8.The contribution rates of the first three principal components are 57.98%, 13.34% and 13.15%, respectively, and the cumulative contribution rate reaches 90.47%, which is the largest when combined with the results of the fragmentation diagram (Fig 8) results, these three principal components have the largest contribution.

**Table 6:** Eigenvalues of the correlation coefficient matrix for the Langyashan study.

Ingredients	Eigenvalues	Contribution rate%	Accumulated contribution rate%
1	5.798	57.983	57.983
2	1.934	19.336	77.319
3	1.315	13.148	90.467
4	0.607	6.072	96.539
5	0.295	2.949	99.488
6	0.051	0.512	100.000
7	1.261E-16	1.261E-15	100.000
8	6.599E-17	6.599E-16	100.000
9	−1.06E-16	−1.06E-15	100.000
10	−4.34E-16	−4.344E-15	100.000

The matrix was rotated by the maximum variance method (Table 7). The results can be concluded that soil total nitrogen, soil organic carbon, soil water content, soil porosity and elevation loadings are large in the first principal component, which is the main characterisation index of the first principal component; soil water content, average diameter at breast height (ADB), elevation and soil total phosphorus are the main loadings in the second principal component; and the third principal component has large loadings of the indicators soil water content, soil total potassium and soil total phosphorus. The highest loadings were 0.96, 0.96 and 0.82 for soil total nitrogen, soil organic carbon and soil water content in the first principal component, 0.96 and 0.71 for soil mean diameter at breast height and elevation in the second principal component, and 0.82 for soil total phosphorus in the third principal component.

**Table 7:** Factor rotation results for the Langyashan study area.

Factor	Ingredients		
1	2	3
Soil total nitrogen	0.958	0.188	0.050
Soil organic carbon	0.956	0.173	−0.091
Soil Bulk Weight	−0.956	−0.183	−0.082
Soil moisture content	0.819	0.305	0.288
Soil potassium	−0.799	−0.012	0.567
Soil porosity	0.794	0.246	0.153
Average diameter at breast height	0.107	0.964	0.014
Elevation	0.492	0.791	−0.132
Stand height	0.069	0.294	−0.899
Soil phosphorus	0.393	0.340	0.817
Variance contribution/%	57.983	19.336	13.148
Cumulative contribution/%	57.983	77.319	90.467

**Fig 9:**
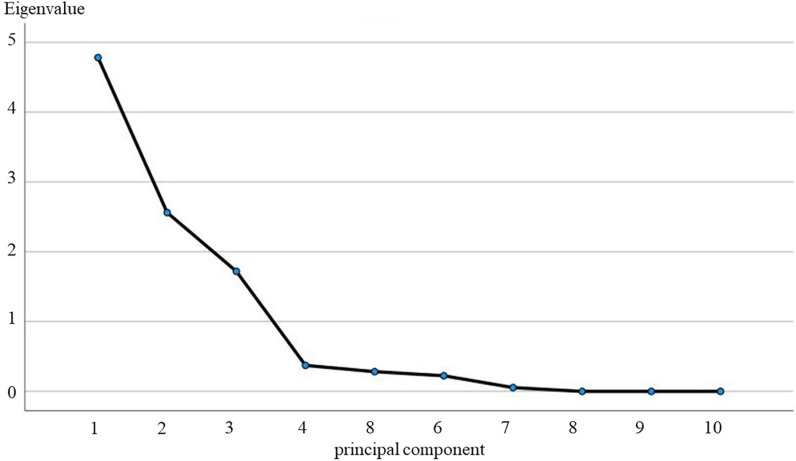
Gravel map of principal component analysis for the Zunhua study area.

Using SPP22.0 software to calculate the 10 indicator factors in the northern karst Zunhua study area, aiming to obtain the contribution rate and cumulative contribution rate of each principal component, in which the eigenvalues of principal components and the contribution rate results are shown in Table 8 and Fig 9. The contribution rates of the first three principal components are 47.83%, 25.62% and 17.20%, respectively, and the cumulative contribution rate reaches 90.66%, which is the largest when combined with the results of the fragmentation diagram (Fig 9) results, these three principal components have the largest contribution.

**Table 8:** Eigenvalues of the correlation coefficient matrix for the Zunhua study area.

Ingredients	Eigenvalues	Contribution rate%	Accumulated contribution rate%
1	4.783	47.833	47.833
2	2.562	25.620	73.453
3	1.720	17.204	90.657
4	0.373	3.728	94.385
5	0.283	2.826	97.211
6	0.224	2.243	99.454
7	0.055	0.546	100.000
8	3.728E-17	3.728E-16	100.000
9	2.854E-19	2.854E-18	100.000
10	−1.52E-16	−1.52E-15	100.000

The matrix was rotated by the maximum variance method (Table 9). The results can be concluded that soil total nitrogen, soil organic carbon and soil porosity loaded the most in the first principal component with 0.95, 0.90 and 0.88, which are the main characterisation indexes of the first principal component; soil water content and soil total phosphorus were the main loadings in the second principal component with the values of 0.92 and 0.83; and elevation and stand height indexes loaded the most in the third principal component with the values of 0.94 and 0.67.

**Table 9:** Zunhua study area factor rotation results.

Factor	Ingredients		
1	2	3
Soil total nitrogen	0.946	−0.149	0.240
Soil Bulk Weight	−0.932	−0.039	−0.297
Soil organic carbon	0.896	−0.215	−0.077
Soil porosity	0.877	0.210	−0.008
Soil water content	−0.301	0.922	0.056
Average diameter at breast height	−0.201	−0.877	−0.143
Soil total phosphorus	0.029	0.833	−0.466
Elevation	−0.095	0.080	0.937
Soil potassium	−0.570	0.028	−0.791
Stand height	0.487	−0.494	0.671
Variance contribution/%	47.833	25.620	17.204
Cumulative contribution/%	47.833	73.453	90.657

#### 3.2.2. Mechanisms of ecological product value realisation for afforestation in different karst desertification control areas.

Due to the different ecological environments, humanistic conditions and economic activities in different regions, the mechanisms and paths for realising the value of EP are also diversified. In order to explore in depth the paths and mechanisms for realising the value of afforestation under different KDC contexts, this section first analyses in detail the influencing factors of the value of EP under different KD environments, and then carries out a comparative study on the paths and mechanisms for realising the value of different EP. Through this process, we clearly identify the strengths and weaknesses of each study area in terms of ecological resources, and discover the shortcomings in the current value realisation mechanisms. These studies not only help us to understand the value transformation process of EP in KDC more comprehensively, but also provide a solid theoretical foundation and more perfect guidance on the path and mechanism of value realisation to further promote the value realisation of high-quality EP.

**(1) Analysis of soil factors affecting the dominant functional ecological products of afforestation in different karst desertification control areas:** The growth and maintenance of forests are closely dependent on the soil and its site conditions, and the interactions between these two factors have a decisive influence on the state of forests. Therefore, an in-depth investigation of the interactions between soil physical and chemical properties and site conditions is essential to accurately assess the state of forests and identify their potential for EP supply.

By applying the statistical method of principal component analysis, we systematically analysed the relationship between soil and forest status in different study areas. In the Shibing study area, stand height, average diameter at breast height, organic matter content, and total nitrogen content of afforestation for KDC were identified as the main drivers influencing the value of EP regulating services. In contrast, in the Langyashan study area, total nitrogen, organic matter, and soil moisture content were identified as key factors influencing the value of EP regulation services. It is worth noting that there was a highly significant positive correlation between climate regulation products and organic matter content in this area, and also a significant positive trend with total nitrogen content. For the Zunhua study area, total nitrogen, organic matter, and soil porosity were the dominant factors influencing the value of EP regulation services. In particular, the value of atmospheric purification services showed highly significant positive correlations with both total nitrogen and organic matter contents, a finding that is important for understanding the contribution of soil nutrients to forest ecological service functions.

**(2) Comparative analysis of the mechanisms of ecological product value realisation in afforestation:** In the study area of the Heichong sub-basin in the buffer zone of the Shi Bing Karst World Natural Heritage Site, the operation and development path still lacks systematic guarantee. Currently, there is neither a special trading platform nor unified management by professional cooperatives in the area, and the policy peddling mechanism is not sound, resulting in the realisation of the value of EP in an unstable state. In terms of ecological compensation, the mechanism is not yet perfect, and local residents’ subsidies for forest ecological restoration have basically failed to be implemented, which greatly limits their motivation to participate in ecological protection and restoration. Relying only on policy enforcement to maintain the ecological safety and long-term development of afforestation, it is difficult to effectively stimulate the residents’ enthusiasm for ecological development. Although the Langyashan Sub-watershed Study Area of the Langyashan National Forest Park in Yixian County has shown initial success in its management and development path, the targets are scattered, and there are many types of economic fruit forests, making it difficult to form an influential ecological brand. In terms of ecological compensation, there is almost no mechanism for residents to participate in afforestation to obtain only a one-time labour subsidy, and there is no right of ownership over the afforestation results, which naturally makes it difficult to stimulate their enthusiasm for participation. The Huangtuling sub-watershed study area in the Zunhua National Three-North Protective Forest Project Area has solved the residents’ sales problems through a unified platform for goods stores, and has made use of online sales channels to sell economic fruits, forming the development mode of “Internet + Ecology”. However, policy support for afforestation subsidies is still insufficient, and there is a lack of policy support for fruit farmers to build their own orchards, which makes fruit farmers lack the ability to cope with reduced yields. In addition, due to the lack of an ecological compensation mechanism, the construction of ecological forests in the area has almost come to a standstill, and the only ecological projects are limited to the closed management of the original forests.

Taking an overview of the three study areas, although afforestation have great potential for development, the mechanisms and paths for realising the value of EP are still at the level of traditional commodity trading, and advanced market mechanisms such as forest rights trading and carbon sink trading have not yet been developed. At the same time, the horizontal compensation mechanism between ecological protection areas has not been established, resulting in local residents failing to obtain corresponding economic benefits when providing ecological security for downstream economic development. In general, the three areas have development potential. Value realization mainly depends on traditional trading; new methods are underdeveloped, benefiting few residents. Shibing lacks security and compensation; Langyashan has fragmented goals and little compensation; Zunhua has innovative sales but lacks afforestation support. Mechanisms need improvement..

## 4. Discussion

### 4.1. Formation and optimisation strategies of eco-industry driven by value realisation of afforestation eco-products

**Fig 10:**
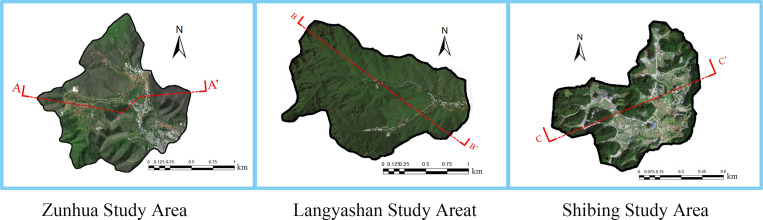
Schematic location of the profile of the design of the ecological industry for afforestation for karst desertification control. Source: Jilin-1 satellite image data (https://www.jl1mall.com/lab/).

For a long time, human society’s perception of ecological resources has often been limited to their “importance” and neglected their “value”, which has led to the protection of ecological resources being constrained only by policies and ethical norms, and lacking deep-seated internal motivation. This cognitive bias directly affects the enthusiasm of human beings to protect ecological resources, which in turn leads to a great waste of resources. In the investigation of mixed-agroforestry ecosystems for KDC, the scientifically optimized mixed-agroforestry system identified by Xiao Jie et al. demonstrates multifaceted benefits. This system not only enhances the productivity of KD lands and mitigates soil erosion and water runoff but also sustains the livelihoods of local farming communities, preserves biodiversity, and delivers critical ecosystem services to the ecologically fragile regions [30]. Similar to mixed agroforestry, afforestation play a pivotal role in ecological restoration and conservation efforts aimed at KDC. They provide a diverse range of services that support human production activities; however, their full potential remains underexplored and underutilized.. Therefore, how to effectively develop the value of afforestation and enhance the enthusiasm of human beings to develop and protect them has become an important issue that needs to be solved urgently. Studies have shown that only when EP providers are able to derive additional benefits from afforestation over other land use types will they have an economic incentive to maintain and improve the ecosystem [31]. This not only helps to improve the conservation efficiency of ecological resources, but also promotes the coordinated development of ecology and economy. By means of the profile form (Fig 10) it is possible to present the optimisation strategy of the EI under the realisation of the value of the EP.

#### (1) Discussion on the optimisation of afforestation resources and ecological industry development strategy in Zunhua study area.

**Fig 11:**
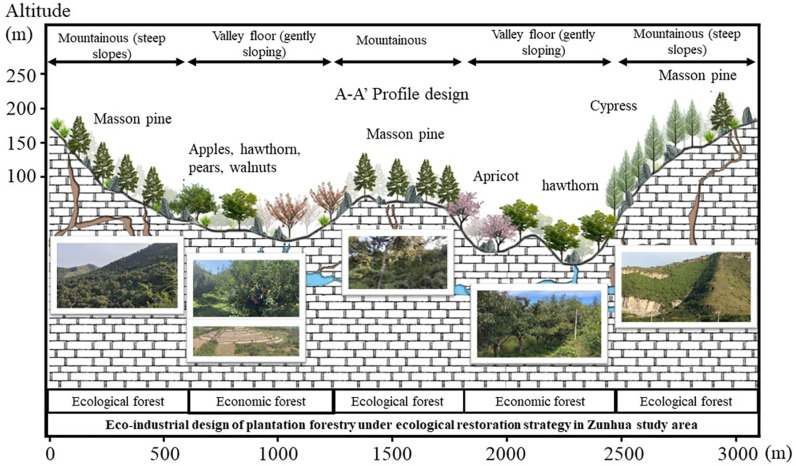
Eco-industrial design of afforestation at different altitudes in the Zunhua study area.

In the Zunhua study area, despite the wide range of economic forest species, the value realisation rate of their development direction is yet to be improved, showing that the economic forest composition in the region has significant development potential. Specifically, the value of EP per unit area was as high as 3,727,600 RMB/hm² for peach and 2,487,800 RMB/hm² for pear, yet the planted area of these two high-value fruit trees was relatively small. In contrast, the EP value per unit area of walnut and nectarine is lower, at 617,500 yuan/hm² and 615,700 yuan/hm², respectively. It is worth noting that although the planted area of nectarine is the largest, its location is not suitable for the development of economic forest. And although peaches and pears are of high value, it is difficult to give full play to their high-value attributes due to the limited planting area. In order to effectively adapt to the needs of realising the value of EP, we propose the following optimisation strategy: in the area above the hillside with large slopes, the existing growth status of oleander and cypress should be maintained, and at the same time, cypress and oleander should be replanted in the open space, so as to lay the foundation for the formation of large-scale ecological regulation services in the later stage. As for the foothill area with gentle slope, the planting area of walnut and apple should be reasonably controlled, and the planting area of peach and pear should be increased, so as to form an ecological industrial model with large-scale and high EP value. Specifically, priority can be given to the development of representative tree species such as peach and pear, supplemented by apricot, hawthorn and apple, etc., to build a scientific and reasonable direction for the development of afforestation EI. The aim is to form a unique ecological brand in the region, and then enhance the economic income of farmers through the value-added effect of EP (Fig 11).

#### (2) Discussion on the optimisation of afforestation resources and the development strategy of ecological industry in Langyashan study area.

**Fig 12:**
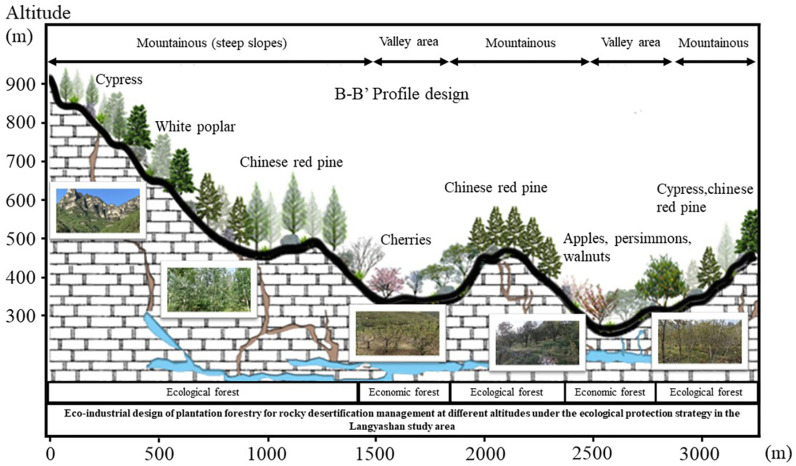
Eco-industrial design of afforestation at different altitudes in the Lang Ya Shan study area.

lthough cypress and persimmon are the main afforestation species in the Langyashan study area, they are not the highest representatives of their EP value. Cherry was notable for its EP value per unit area of 2,168,600 Yuan/hm², however, its afforestation area was relatively limited. On the contrary, oil pine has the lowest EP value per unit area, only 613,200 Yuan/hm². In the gently sloping area suitable for economic forest development, walnut also had a lower EP value per unit area of 633,300 Yuan/hm². Given the limited area of gentle areas suitable for economic forest development in the Langyashan study area, targeted optimisation strategies are needed to maximise the output of regional EP value. Firstly, the planting area of walnut should be reduced, and the planting area of cherry should be increased instead, in order to construct an EI development direction dominated by characteristic material products. This strategy aims to form a regionally unique ecological brand, thereby enhancing the market competitiveness of EP and improving the economic returns of farmers (Fig 12). In addition, priority should be given to the protection of existing ecological forest resources, such as cypress, oil pine and aspen, in the region of steep slopes of high mountains. These tree species play a key role in maintaining regional ecological balance and biodiversity, and their ecological value should not be ignored. Through the implementation of scientific protection and management measures, the continued healthy development of these ecological forest resources can be ensured, providing a solid guarantee for the ecological security and sustainable development of the region.

#### (3) Discussion on the optimisation of afforestation resources and ecological industry development strategy in Shibing Research Area.

**Fig 13:**
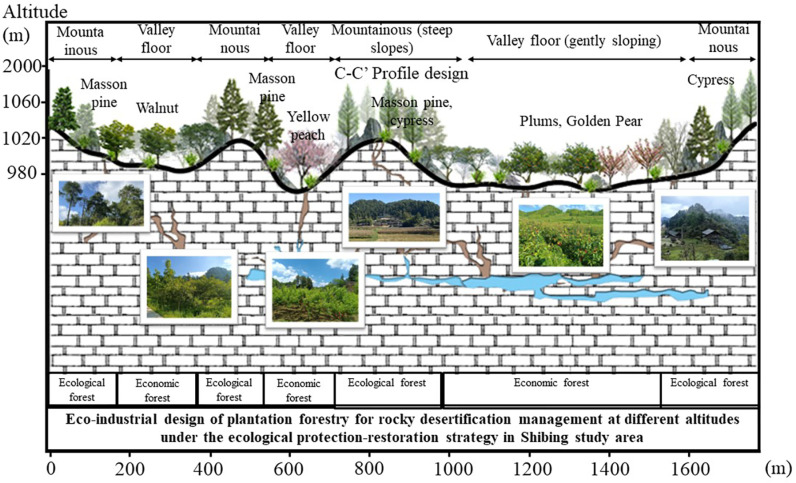
Eco-industrial design of afforestation at different altitudes in the Shibing study area.

Shibing study area has abundant afforestation resources, and its ecological forest and economic forest resources are relatively reasonably distributed. Among them, plum shows high economic value with its EP value per unit area of 1,391,700 Yuan/hm², however, its planting area is still insufficient. In contrast, although the EP value per unit area of horsetail pine is lower, only 680,200 yuan/hm², its extensive planting area plays an important role in maintaining the regional ecological balance. To address this situation, we propose the following optimisation strategies: firstly, the planting area of ponytail pine should be maintained to maintain the existing ecological forest pattern and ensure regional ecological security. At the same time, the planting area of plum should be increased in areas where the terrain is flat and suitable for development in order to realise the potential of its high EP value. Through this strategy, we can establish an EI development model that focuses on both protection and restoration, form a unique regional eco-brand, and thus enhance the market competitiveness of EP and increase the economic income of farmers (Fig 13). In addition, we note that the artificial forestation structure in the Shi Bing study area is relatively simple, and the understory space advantage has not been fully utilised. In order to further increase the comprehensive value of EP, we suggest that we learn from the successful experience of planting Chinese herbal medicines under the forest in Baili Township and develop an EI of under-forest economy. Through reasonable planning and development, we can make full use of the understory space and improve the land utilisation rate, as well as promote the coordinated development of ecology and economy to maximise the ecological value.

#### (4) Analysis of Commonalities in Industrial Development and Optimisation Strategies of the Three Study Areas.

After an in-depth analysis and comprehensive design of the three study areas, we found several common features in industrial development in these areas and proposed corresponding optimisation strategies. Firstly, all three study areas have the problems of simple afforestation structure and under-utilisation of understorey space. In order to fully exploit the potential of understorey space, we suggest drawing on the successful experience of understorey cultivation of traditional Chinese medicinal herbs and understorey aquaculture to develop understorey economic and EI, so as to realise the rational development of ecological value. Secondly, all three study areas lack effective industrial models in realising the value of ecological regulation service products. In order to promote the realisation of EP value, we suggest centralised management of property rights through market means, government policies, and residents’ cooperatives. At the same time, the current national forest rights trading market and carbon sink trading market and other EI models should be used to promote the transformation and realisation of EP value. Finally, all three study areas have deficiencies in the ecological compensation mechanism for afforestation ecological protection and restoration. In response to this problem, we put forward the following suggestions: the natural heritage-based Shibing study area should establish a state-led vertical compensation mechanism to ensure the sustainability of ecological protection and restoration; the Langyashan study area should form a cross-city horizontal compensation mechanism, especially cooperation between the upstream and downstream of Baiyangdian, in order to achieve the common protection of the regional ecological resources; and the Zunhua study area should set up a water source The Zunhua study area should establish a horizontal financial subsidy mechanism within the city between upstream and downstream to ensure the stability and improvement of the ecological environment of the water source. In summary, through targeted optimisation strategies and industrial model innovation, the three study areas are expected to achieve sustainable economic development while protecting the ecological environment.

### 4.2. Ecological industrial design

As a key achievement in the management of KD, afforestation plays an irreplaceable role in protecting and improving the ecological environment. Therefore, while ensuring environmental benefits, it is particularly important to develop the afforestation EI and guarantee the long-term sustainable development of afforestation resources in KDC areas. In their research on forest ecosystem functions within the context of KDC, Xiong Kangning et al. highlighted that the establishment of germplasm resource banks and the implementation of vegetation adaptation enhancement strategies can significantly improve the functional traits of diverse vegetation types [32]. These improvements, in turn, optimize the soil microbial environment, enhance soil nutrient conditions, and promote vegetation growth and development. Furthermore, Deng Xuehua et al. demonstrated that investigating the trade-offs and synergies among forest ecosystem services in KDC not only provides a theoretical foundation for optimizing ecosystem functions but also offers valuable insights for enhancing the stability of forest ecosystems [33]. Nevertheless, the traditional forestry industry often faces challenges such as low production efficiency and limited sustainability in its operations [34]. Consequently, the rational development and utilization of forest resources, tailored to the specific conditions of local areas, are of paramount importance. As an area without major ecological reserves and with a large proportion of economic forests, the Zunhua study area has a relatively large space for landscape adjustment of afforestation. In view of this, this study takes Zunhua study area as an example, and discusses in depth the design ideas of the EI of afforestation. By optimising the planar spatial layout and three-dimensional spatial structure of afforestation, it aims to enhance the supply capacity of afforestation EP, and thus give full play to the ecological advantages of the study area. This strategy not only helps to maximise the value of EP, but also drives the vigorous development of EI. At the same time, the development of the industry can in turn promote the in-depth development of EP, forming a virtuous circle in the management of KD.

#### 4.2.1. Ecological industry design principles.

When planning and constructing afforestation eco-industries, it is crucial to establish a set of design principles to ensure the rational layout, efficient management and long-term sustainable development of the forestry industry. These principles aim to maximise the synergistic growth of economic, social and environmental benefits.

**(1) Principles of sustainable development:** The design of the afforestation EI should be firmly grounded in the concept of sustainable development, with a clear focus on long - term benefits. In practical terms, this requires a meticulous assessment of resource availability and demand. For instance, when planning tree - planting activities, forest managers need to consider the growth rate of different tree species, soil fertility, and water availability. By carefully selecting tree species that are well - adapted to the local environment and have a sustainable growth cycle, over - exploitation of resources can be avoided. Additionally, in the process of forest product extraction, a quota system should be implemented. For example, for timber harvesting, only a certain percentage of mature trees should be cut each year, ensuring that the forest ecosystem has enough time to regenerate. This approach guarantees the stable growth of the forestry economy and the continuous improvement of the environment, maintaining a balance between economic development and ecological protection.

**(2) Principle of win-win co-operation:** To achieve the healthy development of the EI, the principle of win - win cooperation must be actively advocated and effectively implemented. The government can play a leading role by formulating favorable policies, such as providing tax incentives for eco-friendly forestry enterprises. This encourages industries to invest in sustainable forestry development. Non - governmental organizations can contribute by conducting research on forest - related ecological and social issues, providing valuable data and recommendations for decision - making. The community, on the other hand, can be involved in forest protection and eco-tourism activities. For example, local communities can be trained to become tour guides for eco-tourism projects in the forest areas, which not only creates job opportunities for them but also promotes the development of the local economy. By encouraging all parties to participate and form a strong synergy, the prosperity of the EI can be jointly promoted, leading to sustainable economic, social, and environmental development.

**(3) The principle of combining artificial intervention and natural succession:** In the design of afforestation EI, a careful consideration of the organic combination of artificial intervention and natural succession is essential. First, foresters need to conduct in - depth ecological surveys of the area. Based on the understanding of local species composition, climate, and soil conditions, artificial intervention can be carried out in a targeted manner. For example, in areas where the natural forest has been damaged due to human activities, artificial seeding or tree - planting of native species can be carried out to accelerate the restoration process. However, this intervention should be carried out in strict accordance with natural laws. For instance, when introducing new tree species, their potential impact on the existing ecosystem, such as competition for resources and possible invasive effects, must be carefully evaluated. By combining artificial intervention with natural succession, not only can the efficiency of forest management be improved, but also biodiversity can be effectively protected, and ecological balance can be maintained.

**(4) Principle of comprehensive utilisation of resources:** To achieve efficient use of resources and minimize waste, the design of the EI of afforestation must adhere to the principle of comprehensive use of resources. In the forest, in addition to timber, non - timber forest products such as wild fruits, mushrooms, and medicinal plants can be exploited in a sustainable manner. For example, forest - based food processing industries can be developed to process wild fruits into jams, juices, etc., adding value to these products. At the same time, ecological services such as carbon sequestration, water conservation, and biodiversity protection can be monetized through mechanisms like carbon trading. By optimizing the allocation of resources, for example, using forest waste for biomass energy production, and improving the overall utilisation efficiency, sufficient resources can be provided for economic development. Moreover, this approach helps to reduce the negative impacts on the environment associated with resource extraction and waste generation.

#### 4.2.2. Ecological Industry layout discussion.

**Fig 14:**
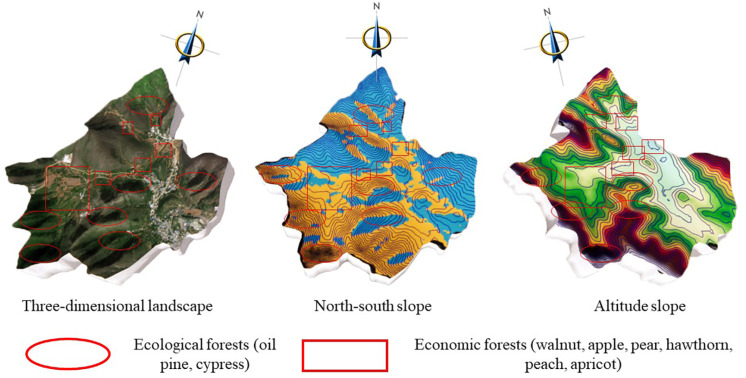
Slope direction and elevation analysis of afforestation in the Zunhua study area. Source: Jilin-1 satellite image data (https://www.jl1mall.com/lab/).

The vegetation distribution in the Zunhua study area presents unique geographical characteristics. In a comprehensive examination of the distribution of slope directions (Fig 14), the distribution of afforestations on the east and west slopes showed a similar trend, while the difference between the north and south slopes was particularly significant. After careful analysis, we found that the vegetation on the northern slopes in the study area grew vigorously, while the southern slopes were mainly dominated by shrubs and herbaceous plants, interspersed with a small number of low-growing cypresses, which, however, grew relatively poorly. Based on the above observations, we further explored the ecological mechanism causing this difference. After analyses, we deduced that this was due to climatic factors specific to the northern region. Specifically, the effect of evapotranspiration in the northern region significantly outweighs the effect of photosynthesis in this region. For the southern slopes of the mountain, evaporation tends to be greater than precipitation due to abundant sunlight, resulting in water stress and thus poor plant growth. In contrast, the northern slopes of the mountain, with less sunlight and less evapotranspiration, provided more favourable conditions for vegetation growth, and thus the vegetation grew better naturally. This finding not only provides important clues for us to understand the ecological mechanism of vegetation distribution in the Zunhua study area, but also provides a scientific basis for future ecological conservation and restoration work in the area.

**Fig 15:**
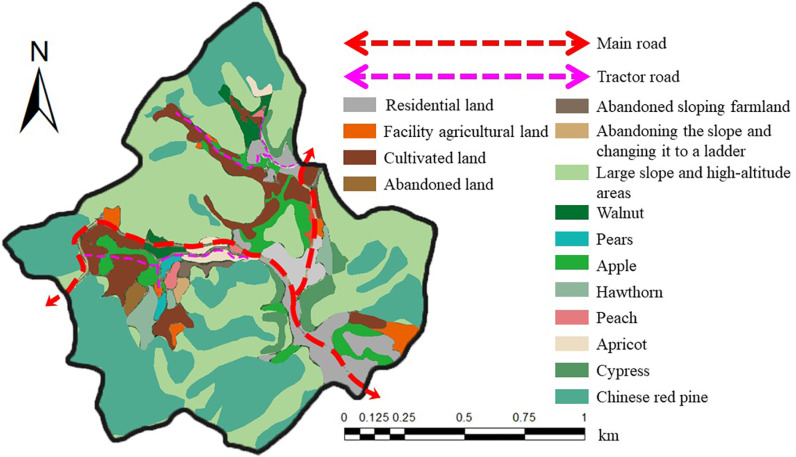
Analysis of the layout of the afforestation industry in the Zunhua study area. Source: Jilin-1 satellite image data (https://www.jl1mall.com/lab/).

In the elevation landscape of the Zunhua study area (Fig 15), we observed that the afforestation showed significant vegetation distribution characteristics at different terrain levels. Specifically, the hillside area is mainly distributed with ecological forests, which are mainly composed of oil pines and cypresses, and provide important ecological service functions for the local ecosystem. On the other hand, the top part of the mountain is mainly composed of shrubs, whose diverse species enrich the ecological landscape. Further observation reveals that in the area below the mountainside, the afforestation are transformed into mainly economic forests. These economic forests mainly include fruit trees such as walnut, apple, pear, hawthorn, peach and apricot, which not only bring considerable economic benefits to local farmers, but also add diversity to the ecological environment. In the valley floor area, which is the flattest terrain, we noticed that there are mainly arable lands distributed here. As an important agricultural production base in the study area, these arable lands provide abundant food and agricultural products for local residents, and at the same time provide important support for the balance and stability of the entire ecosystem. This vegetation distribution dictates the ecological industrial layout. The ecological forests on the north slope and hillside are apt for the development of eco-tourism and forest - based economy. The economic forests beneath the hillside facilitate the growth of fruit - related industries. The arable land on the valley floor can be utilized for green and organic agriculture, thus forming a comprehensive eco-industrial system.

#### 4.2.3. Ecological industrial design.

**Fig 16:**
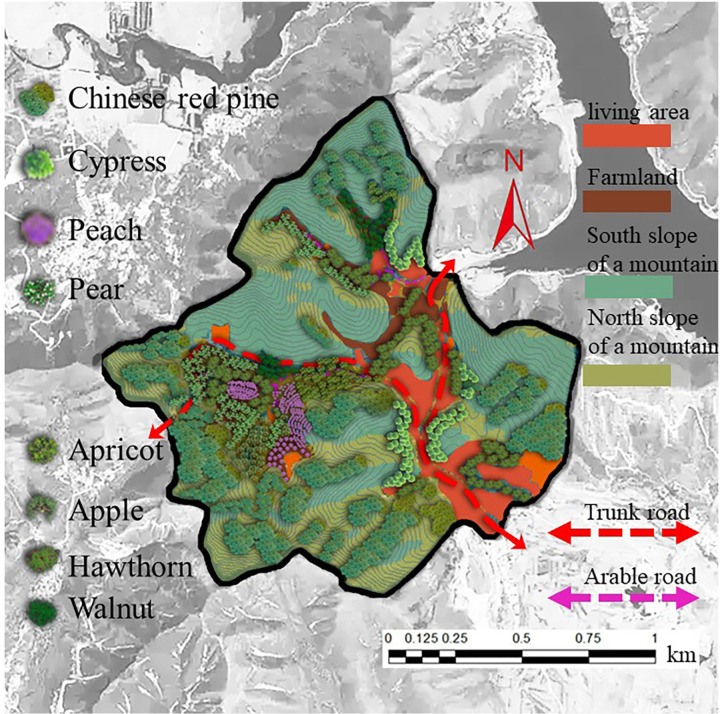
Spatial design of the afforestation eco-industry plan in the Zunhua study area. Source: Jilin-1 satellite image data (https://www.jl1mall.com/lab/).

**(1) Ecological industry planning for afforestation:** The Zunhua study area exhibits a distinctive vegetation distribution pattern. A Y - shaped main road, along with two mechanized roads, connects the areas conducive to economic forestry, effectively meeting the requirements for the development of the EI. Based on the previous analyses of the vegetation distribution and ecological mechanisms in the study area, this subsection aims to provide an exhaustive spatial planning for the afforestation EI in the region (Fig 16).

In terms of ecological forest protection, we adhere to the principle of protection to ensure that the natural recovery process of ecological forests is not interfered with and necessary replanting work is carried out to maintain their ecological stability and biodiversity. For the areas suitable for planting economic forests, we firstly set out to integrate scattered tree species with low EP value (such as walnuts, apples and hawthorns), and concentrate resources for large-scale planting. At the same time, we will not expand the planting area of these species in the future development to optimise the allocation of resources and ensure the sustainable development of the ecological economy. For high-value EP types of tree species, we will, on the basis of the existing foundation, actively develop the surrounding abandoned land suitable for planting, and plant these high-value economic forests on arid and barren plots with low food production. Specifically, in the southeastern region of the study area, we will use dry and barren land to plant peach, pear and apricot trees on a large scale, which will be combined with the existing apple and hawthorn orchards to form a high-yield, high-efficiency afforestation ecological industrial agglomeration. Such a layout is not only convenient for standardised management, but also conducive to the development of the picking industry, increasing farmers’ income. In the northern valley, we will continue to develop the original walnut industry, and at the same time use the spare valley bottom area to build a pear tree industrial park. Such planning aims to make full use of land resources and promote the diversification of industries, while ensuring a harmonious symbiosis between ecology and economy.

In summary, the spatial planning of afforestation EI in the Zunhua study area aims to realise the dual benefits of ecology and economy, and to promote the sustainable development of the area through scientific planning and rational layout.

**Fig 17:**
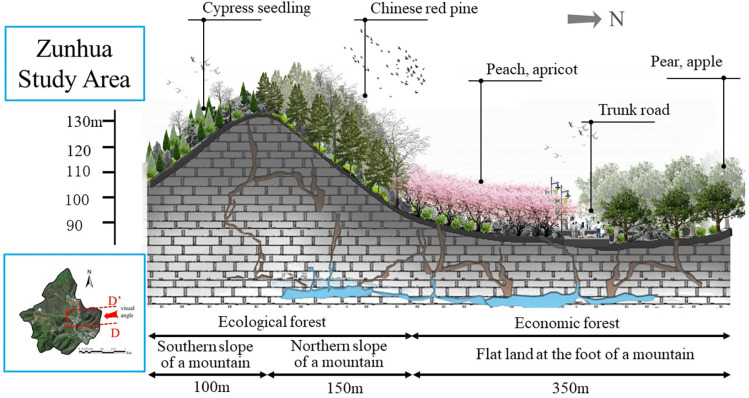
Spatial design of afforestation eco-industry façade in Zunhua study area. Source: Jilin-1 satellite image data (https://www.jl1mall.com/lab/).

**(2) Ecological Industry refinement design:** In the Zunhua study area, the relatively gentle topography leads to limited variation in the spatial vertical distribution of afforestation. However, despite the low topographic relief, factors like water stress and slope exert a significant influence on afforestation distribution, thereby affecting the distribution pattern of EI values. Consequently, when devising eco-industries, elements such as slope orientation and mountain location (including hilltop, hillside, and foothill) must be comprehensively considered to guarantee the scientific planning and rational distribution of eco-industries (Fig 17).

(1) Design based on different slope positions

In the eco-industrial planning of the Zunhua study area, the design of artificial forestation on diverse slope positions is of utmost importance.

Hilltop Area: Retaining the original shrubs and herbaceous plants, we replanted drought - and cold - resistant cypress trees. This approach enhances the ecological stability and adaptability of the hilltop ecosystem. The original vegetation provides a foundation for the new plantings, and the cypress trees, with their resilience to harsh conditions, contribute to the long - term sustainability of the area.
Hillside Area: Building on the existing oil pine and cypress forests, additional cypresses and oil pines were replanted in the arboreal and shrub layers. This action enriches the vegetation structure, increases species diversity, and improves the overall ecological value. A more complex vegetation community can better perform ecological functions such as soil and water conservation and carbon sequestration.
Foothill Area: Given the relatively flat terrain at the foothill, we opted to plant economic forests with high ecological value, such as peaches, pears, apricots, and apples. This choice enables the realization of a win - win situation in terms of economic and ecological benefits. The economic forests not only yield fruits for economic returns but also contribute to environmental protection through functions like soil stabilization and air purification.

(2) Design based on different slope directions

We also implemented differentiated designs for various slope directions.
Southern Slope: Exposed to direct sunlight and having a high evaporation rate, the southern slope requires special attention. Starting from the catchment line, we first considered planting drought - tolerant cypress trees and gradually extended the planting outward. This strategy ensures the stable growth of vegetation by effectively managing water resources. The cypress trees’ ability to withstand drought helps them survive in the water - stressed environment of the southern slope, and their growth also contributes to reducing soil erosion.
Northern Slope: Based on the original afforestation, we further expanded the ecological forest on the northern slope. Simultaneously, in the gentler terrain, economic forests like peaches and pears were planted to enrich the ecosystem’s diversity according to local conditions. The cooler and moister environment on the northern slope is suitable for the growth of both ecological and economic forest species, promoting a more balanced and diverse ecosystem.

Moreover, in the understorey space of ecological forests such as horsetail pine and cypress, we planned diversified eco-industries, including the cultivation of wild mushrooms and the planting of Chinese herbs. This initiative not only effectively utilizes the understorey space, improving land utilization efficiency but also enriches the eco-industrial structure, promoting the virtuous cycle and sustainable development of the ecosystem.

In summary, the design strategy of the EI for afforestation on different slopes and slope directions in the Zunhua study area fully takes into account natural factors such as topography, climate, and vegetation, as well as human factors like economic and social aspects. The aim is to achieve the coordination and integration of ecological and economic benefits, providing robust support for the region’s sustainable development.

## 5. Conclusions

(1) **The value of fruit products, soil conservation, water conservation, atmospheric purification, carbon sequestration and climate regulation of afforestation in the management of karst desertification has been revealed; the value of ecological products of afforestation per unit area and their ecological advantages have been explored in each study area.**

While forest ecosystems provide material products, the value of their ecological regulating products is particularly prominent, which has been clearly confirmed in the research of Zhao Tongqian et al. [35]. In the practice of KD management, afforestation ecosystems have shown their unique ecological value and become a typical representative in this field.

Through on - site investigations and data analysis, the following data were derived for each study area (Table 10). we found that the value of ecological regulation services occupies a significant position in the total value. In the Shibing study area, although the fruit value of afforestation reached 176,600 yuan, its soil conservation value was as high as 9,041,000 yuan, and its water conservation value even reached 12,926,800 yuan. In addition, the value of ecological regulation services such as atmospheric purification, carbon sequestration and climate regulation also reached 14,500,000 yuan, 342,800 yuan and 23,124,900 yuan respectively. Meanwhile, the value of cultural services in the area should not be neglected, reaching 246,300 yuan. In the Wolverine study area, the value of fruits from artificial forestation was 1,576,600 yuan, while the value of ecological regulation services such as soil conservation, water conservation, atmospheric purification, carbon sequestration and climate regulation reached 3,147,200 yuan, 1,579,900 yuan, 15,500,000 yuan, 290,200 yuan and 40,637,900 yuan, respectively. In addition, the value of cultural services in the region was 145,900 yuan. The afforestation in the Zunhua study area also demonstrated significant ecological regulation value. Its fruit value was 5,321,700 yuan, while the values of ecological regulation services, such as soil conservation, water conservation, atmospheric purification, carbon sequestration and climate regulation, reached 6,245,800 yuan, 568,900 yuan, 140,000 yuan, 274,400 yuan and 35,279,800 yuan, respectively. In addition, the value of cultural services in the region is $13.8 million.

**Table 10:** Value of ecological products from afforestation for karst desertification control.

Study area	Material products (million yuan)	Soil conservation (million yuan)	Water conservation (million yuan)	Atmospheric purification (million yuan)	Carbon sequestration (million yuan)	Climate regulation (million yuan)	Cultural services (million yuan)
Shibing	17.66	904.10	1292.68	1.45	34.28	2312.49	24.63
Langyashan	157.66	314.72	157.99	1.55	29.02	4067.91	14.59
Zunhua	532.17	624.58	56.89	1.40	27.44	3527.98	13.80

Summarising the above data, we can clearly see that in karst KDC, afforestation ecosystems not only provide rich material products for the local area, but also play a great role in ecological regulating services. These ecological regulation services not only guarantee the health and stability of the ecosystem, but also provide strong support for the sustainable development of human society. Therefore, in future ecological restoration and protection, we should pay more attention to and give full play to the ecological regulation function of afforestation ecosystems.

**Fig 18:**
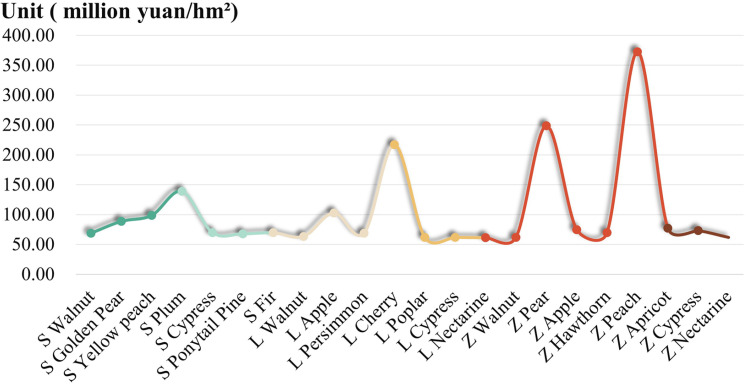
Value of ecological products of afforestation per unit area for Karst Desertification Control.

Examined from the perspective of EP value per unit area of afforestation (Fig 18), all three study areas showed the phenomenon that the EP value per unit area of economic forests occupied a prominent position. Specifically, the value per unit area of peach forest in Zunhua study area was the most prominent (RMB 3,727,600/hm²), surpassing cherry forest in Langyashan study area (RMB 5,168,600/hm²) and plum forest in Shibing study area (RMB 1,391,700/hm²). This trend highlights the significant ecological and economic advantages of different study areas in specific economic forest types. Further analysing in depth the value of EP per unit area of afforestation in the three study areas, we found that: plum forests (1,391,700 yuan/hm²) led the way in Shibing study area, followed by yellow peach (984,500 yuan/hm²) and golden pear forests (885,600 yuan/hm²); while cherry forests (2,168,600 yuan/hm²) led the way in the Wolongashan study area, and apple forests (1,028,900 Yuan/hm²) followed by persimmon forest (687,000 Yuan/hm²) in the third place; in Zunhua study area, peach tree (3,727,600 Yuan/hm²) and pear tree (2,487,800 Yuan/hm²) ranked in the top two places respectively, and apricot tree (773,100 Yuan/hm²) ranked in the third place. Here, ecologically superior forest types are determined on the basis of the value of EP per unit area. The economic forests in Shibing study area showed significant advantages in plum forests, while in the ecological forests, cypress forests occupied a dominant position; the economic forests in Woluyashan study area showed advantages in cherries and apples, but the ecologically dominant forest types in the ecological forests were not obvious; among the economic forests in Zunhua study area, peach and pear trees showed significant ecological advantages, while among the ecological forests, cypress forests likewise occupied a dominant position in the ecological forests. These findings not only provide important references for ecological restoration and industrial development in the study areas, but also provide empirical evidence to understand the role of different forest types in ecosystem service values.

(2) **We hereby propose a strategy for optimizing the afforestation EI in the context of Karst Desertification Control. Vertically, from higher to lower altitudes, the industry layout should consist of ecological forests, an ecological - economic forest transition zone, and economic forests. In this framework, choosing dominant tree species is key to optimizing the structure of both ecological and economic forests. This demands adjusting the proportion of the two forest types based on the region’s unique ecological features. For economic forests, species selection should prioritize those well - adapted to local conditions and with high economic returns, to help build a distinct ecological - label brand.**

**Fig 19:**
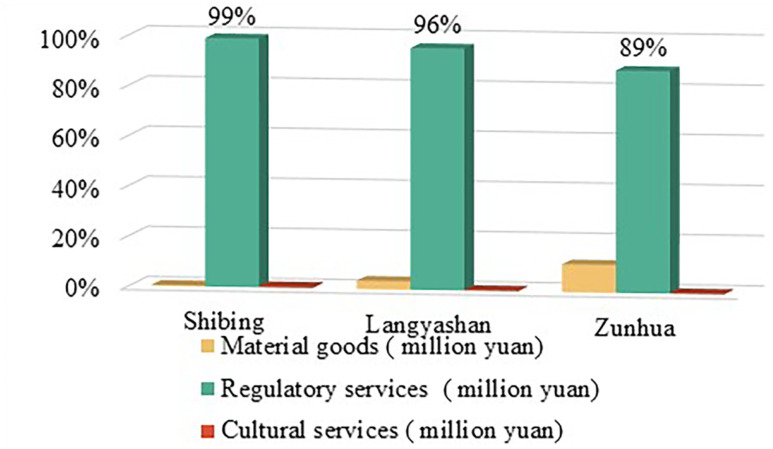
Differences in the value share of ecological products in the three study areas.

With the in-depth implementation of ecological projects such as KDC, afforestation has been significantly promoted and developed. In this context, the development of afforestation EI, and then promote the realisation of the value of EP, has become a key path for ecological restoration and sustainable development. The study by Chen Xingliang et al. [36] points out that the development of afforestation EI needs to shift from the pure pursuit of increasing the quantity of EP to the strategic shift of pursuing high-quality comprehensive product output. In the value composition of afforestation EP, the value of regulating services occupies a dominant position (Fig 19). Particularly noteworthy is that ecological forests account for the vast majority of the contribution to the value of regulating services, while economic forests only account for a smaller share. This finding emphasises the importance of protecting existing ecological forest resources and suggests that we need to create conditions for positive succession in afforestation ecosystems by implementing measures such as logging and grazing bans.

In the same ecosystem, there are differences in the value of EP in different areas. In the three study areas, for example, the value of EP per unit area of economic forests varied and showed differences in specific ecological functions. For example, in the Shibing study area, the value of EP per unit area of plum forest was higher than that of horsetail pine forest, and overall, the value of economic forest exceeded that of ecological forest. Further analysis revealed that although the planted area of golden pear was larger than that of plum, the EP value per unit area of plum was higher, which revealed the correlation between the EP value of economic forests and the planted area. Therefore, from the perspective of EI optimisation, we can consider increasing the planting area of plums and reducing the area of other economic forest types accordingly. In the Langyashan study area, cherries and apples have the highest EP value per unit area. This suggests that we should give priority to increasing the planting scale of cherries and apples while controlling the development of persimmons in the EI layout. And in Zunhua study area, although the unit area value of peach is higher than that of hawthorn, hawthorn, as a speciality industry in the area, changing the industrial structure may be a more effective way out.

Comparing the value of EP per unit area in the three study areas, we find that Zunhua study area is the highest, followed by Langyashan, and finally Shibing. This result is related to the fact that the rainfall in the northern region is smaller than that in the southern region, as well as the production of mericarps and the supply and demand in the market. However, in the analysis of the value of regulating services, the results showed the opposite trend, i.e., Shi Bing study area was the highest, followed by Langyashan Mountain, and Zunhua was the lowest. This reflects the significant influence of natural conditions such as temperature and precipitation on vegetation growth conditions and EP supply [37]. Therefore, for the northern region, it is of more urgent significance to improve the ecological efficiency and promote the optimal layout of afforestation EI to enhance the value of EP of regional economic forests.

## Supporting information

S1 Table Means of sampling data in the Shibing study area. (XLSX)

S2 Table Standard deviations of sampling data in the Shibing study area. (XLSX)

S3 Table Means of sampling data in the Langyashan study area. (XLSX)

S4 Table Standard deviations of sampling data in the Langyashan study area. (XLSX)

S5 Table Means of sampling data in the Zunhua study area. (XLSX)

S6 Table Standard deviations of sampling data in the Zunhua study area. (XLSX)
